# Supplementary material for: The Challenge of Diagnosing Labyrinthine Stroke—A Critical Review
Source: Brain Sci. 2025 Jul 7;15(7):725. doi: 10.3390/brainsci15070725 (PMC12293903; doi:10.3390/brainsci15070725)
Supplement: Supplementary file 1 [file brainsci-15-00725-s001.zip › brainsci-3730562-supplementary.pdf]

Supplementary material – detailed description of the literature search and search results

Methods:

We searched MEDLINE for English language articles (with no limitation applied to the publication year), relying on the following strategy and looking for specific components in all articles: (1) an acute/new onset of symptoms, (2) clinical or radiologic signs of ischemic/hemorrhagic stroke, and 3) involvement of the labyrinth and/or the anterior inferior cerebellar artery (AICA). We then selected a series of textual terms to enter into the search system that would refer to the selected criteria. A manual search of the references of eligible articles was also performed, and we contacted the corresponding authors where necessary. We did not seek to identify the research abstracts from meeting proceedings or unpublished studies. Since the submitted work is a systematic review, ethical approval was not necessary.

MEDLINE Search *(accessed via PubMed at www.ncbi.nlm.nih.gov/pubmed* on May 19th 2025):

(((Acute OR new-onset OR sudden OR rapid OR abrupt) AND (Labryrinthine OR labyrinth OR inner ear OR audiovestibular OR aica OR 'anterior inferior cerebellar artery')) AND (Stroke OR ischemi* OR TIA OR occlusion OR hemorrhag*)) AND eng[la].

Study selection and quality assessment

We used predetermined exclusion criteria and a controlled methodology to select the relevant studies. This was conducted by a single rater (AAT). Only English language articles with original data on human subjects with a labyrinthine stroke, reporting on either vertigo/dizziness/gait ataxia or hearing loss, were included. The search strategy was designed by a clinical investigator with relevant domain expertise in neurology (AAT).

Our search identified 634 unique citations. Of the 634 papers screened, 557 (87.9%) were excluded at the abstract level (see the PRISMA flow chart in Figure 1). We further examined 88 manuscripts at the full-text level (including 11 additional records after a literature search of the included manuscripts). While 75 were considered eligible, 13 were excluded for the following reasons: four did not report on patients with lesions involving the AICA territory or the labyrinth, four did not report on patients with either hemorrhagic or ischemic stroke of the brain or the labyrinth, three did not contain any original data, and two did not report on clinical symptoms, such as vertigo, dizziness, gait imbalance, or hearing loss.

PRISMA 2020 flow diagram: The challenge of diagnosing labyrinthine stroke

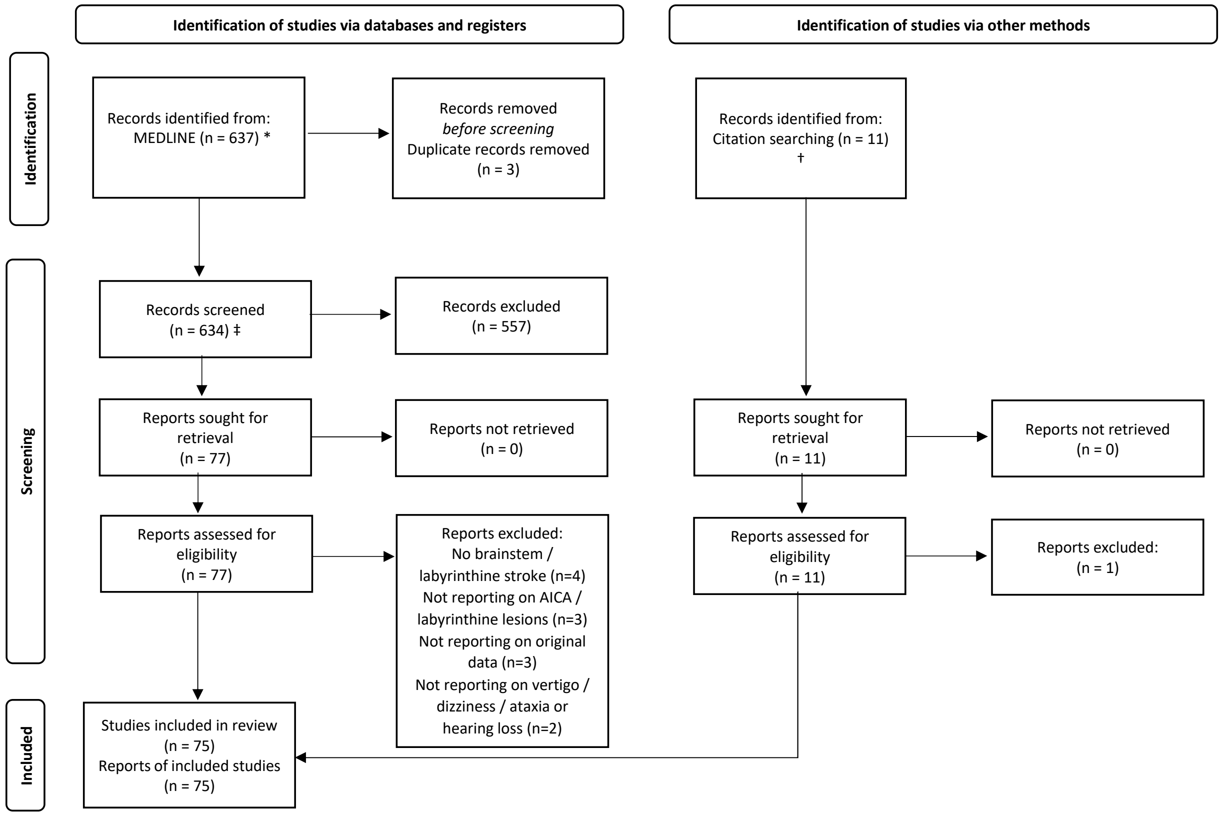

* MEDLINE was accessed via PubMed. † Eleven manuscripts in total were identified through hand search. ‡ Abstracts coded as "yes" or "maybe" by the reviewer were included in full-text review.

Source: Page MJ, et al. BMJ 2021;372:n71. doi: 10.1136/bmj.n71.

Figure legend Figure S1:

PRISMA flow chart (source: [1])

* MEDLINE was accessed via PubMed.

† Eleven manuscripts in total were identified through a hand search.

‡ Abstracts that were coded as either “yes” or “maybe” by the reviewer were included in the full-text review.

Coding schema for abstract and full-text reviews

All of the gathered literature was subject to title/abstract screening by one reviewer (AAT). Full-text screening was then applied to all the citations that were considered eligible or possibly eligible by the reviewer. The reviewer (AAT) then determined whether the full-text manuscripts were eligible and, if not, provided a reason for their exclusion. AAT completed a hand search of the reference lists of selected articles for any additional citations. For the citations identified by the hand search, the full process was repeated iteratively until no additional manuscripts were found for inclusion. A formal review protocol was not registered or posted.

Abstract Review Coding Rules

1) The coding status options are “Yes”, “No”, and “Maybe”. We will review the full text of “Yes” and “Maybe”. The purpose of “Yes” vs. “Maybe” is to look at the kappa values’ agreement on “Yes” vs. “Maybe”.
2) Err on the side of “Maybe” if there is doubt about a “No”; this is more conservative.
3) If there is only a title, exclude it only if you feel confident; otherwise, code it as “Maybe”.
4) Each "No" or "Maybe" should be coded with a reason for its exclusion.
5) The reasons for exclusion are listed below from 0 to 6. Go through them to decide whether any exclusion criteria are met.
6) At the title/abstract screening level, no reason for exclusion is requested.
7) Occasionally, an abstract seems inappropriate for another reason. In such cases, code this as “Other”. There should be a few “Other” codings.

Abstract Reasons for Exclusion

0	Not English	Manuscript is not in English
1	No data	Review paper; no original patient data

2	no dizziness/ataxia or hearing loss	Not reporting on vertigo, dizziness, gait imbalance, or hearing loss
3	Not acute	Not reporting on patients with acute or subacute symptoms, i.e., symptom duration > 14 days
4	No AICA or labyrinth involvement	Not reporting on patients with (presumed) involvement of the AICA territory or the labyrinth
5	Not stroke	Not reporting on patients with underlying ischemic or hemorrhagic stroke
6	Other	Any other reason why the citation should not be included

Full-Text Review Coding Rules

1) Coding status options are “Yes” or “No”.
2) Each "No" should be coded with a reason for its exclusion.
3) Reasons for exclusion are listed below from 0 to 6. Go through them in order from 0 to 6 for each full text, coding the first reason for exclusion only, not multiple reasons for exclusion.
4) A single rater will code the reason for its exclusion.

Full-Text Reasons for Exclusion

0	Not English	Manuscript is not in English
1	No data	Review paper; no original patient data
2	No dizziness/ataxia or hearing loss	Not reporting on vertigo, dizziness, gait imbalance, or hearing loss
3	Not acute	Not reporting on patients with acute or subacute symptoms, i.e., symptom duration > 14 days
4	No AICA or labyrinth involvement	Not reporting on patients with (presumed) involvement of the AICA territory or the labyrinth
5	Not stroke	Not reporting on patients with underlying ischemic or hemorrhagic stroke
6	Abstract only	Published only as an abstract or conference proceeding, or no access to the full-text manuscript

References

1. Page, M.J.; McKenzie, J.E.; Bossuyt, P.M.; Boutron, I.; Hoffmann, T.C.; Mulrow, C.D.; Shamseer, L.; Tetzlaff, J.M.; Akl, E.A.; Brennan, S.E.; et al. The PRISMA 2020 statement: an updated guideline for reporting systematic reviews. *BMJ* **2021**, *372*, n71, doi:10.1136/bmj.n71.
